# Iron Deficiency in Immune-Mediated Inflammatory Skin Diseases: A Missing Link Between Systemic Inflammation, Immunometabolism, and Disease Burden

**DOI:** 10.3390/cells15050478

**Published:** 2026-03-06

**Authors:** Emilia Kucharczyk, Klara Andrzejczak, Karol Biliński, Matylda Korgiel, Małgorzata Ponikowska

**Affiliations:** 1 Faculty of Medicine, Wroclaw Medical University, Wybrzeże L. Pasteura 1, 50-367 Wroclaw, Poland; 2 University Centre of General Dermatology and Oncodermatology, Wroclaw Medical University, ul. Borowska 213, 50-556 Wrocław, Poland

**Keywords:** iron deficiency, immunometabolism, immune-mediated inflammatory skin diseases, iron–skin axis, hepcidin, ferroportin, inflammation, psoriasis, atopic dermatitis

## Abstract

Iron deficiency (ID) has emerged as a pivotal yet underrecognized factor in the pathogenesis of immune-mediated inflammatory skin diseases (IMISDs) such as psoriasis, atopic dermatitis, and hidradenitis suppurativa. Beyond its classical role in erythropoiesis, iron acts as a key modulator of immune cell activity, redox balance, and overall metabolic homeostasis. This review synthesises the latest evidence on the intricate relationship between systemic inflammation, disturbances of iron metabolism, and immunometabolic imbalances that underline the pathogenesis of IMISDs. Findings indicate that chronic inflammation drives functional iron deficiency through IL-6–hepcidin-mediated sequestration of iron, resulting in reduced bioavailability and altered mitochondrial activity in immune and epithelial cells. This imbalance is associated with excessive and chronically enhanced oxidative and inflammatory responses of these cells, further advancing inflammation, anaemia of chronic disease, and disturbances of tissue repair. Moreover, emerging evidence supports an “iron-skin axis,” and suggests that skin cells, particularly epidermal keratinocytes, are actively involved in the regulation of iron pathways. Collectively, these insights position iron homeostasis as a missing link between systemic inflammation, immunometabolic imbalance, and disease burden in IMISDs.

## 1. Introduction

Iron is a trace element essential for the body’s biochemistry; it plays a role in oxygen transport, mitochondrial respiration, DNA synthesis, and the regulation of the immune response [1,2,3]. Besides its conventional role in erythropoiesis, iron is crucial for cellular metabolism, acting as a nutrient, as well as a mediator in the inflammatory microenvironment [3]. There is a fine balance between the nutritional and toxic manifestations of iron in the body. Imbalance in iron homeostasis can be seen in chronic inflammation, leading to conditions of iron deficiency (ID) or inadequacies in iron distribution in the body’s various systems, including the skin [3,4,5].

The immune-mediated inflammatory skin diseases (IMIDs), including psoriasis, atopic dermatitis (AD), and hidradenitis suppurativa (HS), are known for their chronic immune activation and inflammation, in addition to disturbances in glucose and lipid metabolism [4,6]. Growing evidence suggests that these inflammatory conditions are indeed systemic in nature and have implications other than those in the skin. In this context, iron metabolism represents an underexplored yet critical link between systemic inflammation and skin pathology [2,4,7]. Functional iron deficiency (FID) is defined as a state in which total body iron stores are normal or increased, but iron is not adequately available for cellular utilisation due to inflammation-driven sequestration and hepcidin-mediated inhibition of iron export. The resulting restricted iron bioavailability at the tissue and cellular level deprives cells of bioavailable iron despite adequate body stores, contributing to anaemia, fatigue, and impaired tissue repair while sustaining chronic inflammation [5,6,8,9,10,11].

Recent advances in immunometabolism have elucidated how iron availability influences the behaviour of both immune and epithelial cells [12,13,14,15,16]. Iron modulates macrophage polarisation, T-cell differentiation, and keratinocyte function, thereby influencing the overall inflammatory tone of the skin [12,13,14]. Recent findings further indicate that keratinocytes actively contribute to systemic iron regulation via the expression of ferroportin, ferritin, and hepcidin [17,18,19,20]. This interaction represents a new “iron–skin axis” that integrates systemic and local iron handling with immune and metabolic signalling pathways [18,19,21,22].

This review outlines the concept that iron deficiency in immune-mediated inflammatory diseases extends beyond a secondary effect of inflammation and may function as a pathogenic driver linking immune dysregulation, oxidative stress, and metabolic imbalance [12,16,21,23,24]. It further explores key mechanistic pathways through which disturbed iron homeostasis contributes to disease chronicity and systemic comorbidity, highlighting iron metabolism as a potential therapeutic target to reduce overall disease burden [19,20,25,26].

## 2. Iron Regulation

### 2.1. Systemic Iron Regulation

**Figure 1:**
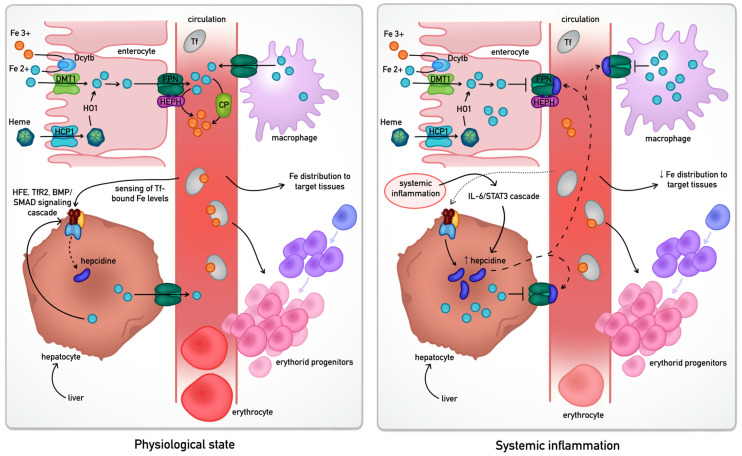
Regulation of systemic iron trafficking in health and inflammatory states [3,6,7]. Dietary non-heme iron, primarily in the ferric (Fe^3+^) form, is reduced to ferrous iron (Fe^2+^) at the apical membrane of duodenal enterocytes by duodenal cytochrome b (Dcytb) and transported into the cell via Divalent Metal Transporter 1 (DMT1). Heme iron is internalised separately (HCP1) and degraded by heme oxygenase-1 (HO-1), releasing iron into the intracellular pool. Iron is exported across the basolateral membrane by ferroportin (FPN) and oxidised by hephaestin or ceruloplasmin, enabling its binding to transferrin for systemic transport and delivery to tissues via transferrin receptor 1 (TfR1). Hepcidin, the principal regulator of iron homeostasis, is produced by the liver in response to systemic iron levels and inflammatory signals. Under physiological conditions, FPN facilitates iron export into the circulation. During systemic inflammation, increased hepcidin induces FPN internalisation and degradation, resulting in reduced plasma iron levels and intracellular sequestration of iron within enterocytes, macrophages, and hepatocytes, thereby restricting iron availability for erythropoiesis and metabolic demands, and contributing to functional iron deficiency. Abbreviations: Dcytb—duodenal cytochrome B; DMT1—Divalent Metal Transporter 1; HCP1—Heme carrier protein 1; HO1—heme oxygenase-1; FPN—ferroportin; HEPH—hephaestin; CP—ceruloplasmin; Tf—transferrin; TfR2—Transferrin Receptor 2.

Systemic iron homeostasis is a tightly orchestrated process primarily managed by the hepcidin-ferroportin axis, which ensures that iron levels in the plasma meet the metabolic demands of tissues while preventing the toxicity associated with iron overload [4]. The human body lacks an active excretory mechanism for iron; therefore, regulation occurs predominantly at the level of intestinal absorption and recycling from senescent erythrocytes [13,20]. The differential regulation of this pathway in physiological conditions versus systemic inflammation is summarised in Figure 1.

Dietary iron—primarily in the ferric (Fe^3+^) form—must undergo reduction to its ferrous (Fe^2+^) state at the apical membrane of duodenal enterocytes. a process mediated by duodenal cytochrome B (Dcytb) or luminal antioxidants like ascorbate [27]. This reduced iron enters the cytosol across the apical membrane of the enterocyte via the Divalent Metal Transporter 1 (DMT1) [28]. Alternatively, heme iron is internalised and degraded by heme oxygenase-1 (HO1) to release inorganic iron into the labile cellular pool [1,2]. Under physiological demand, iron is exported across the basolateral membrane into the portal circulation by ferroportin (FPN), the only known mammalian iron exporter [7]. This efflux is mandatory, coupled with the re-oxidation of iron to its ferric state by the membrane-bound ferroxidase hephaestin in enterocytes, or its soluble homologue ceruloplasmin in macrophages and hepatocytes [2,7,29]. Once in the plasma, iron is sequestered by transferrin, which maintains it in a soluble, non-toxic form and delivers it to tissues—most notably to erythroid progenitors via Transferrin Receptor 1 (TFR1) [3,6,7].

The central iron-stat of this system is hepcidin, a liver-derived peptide hormone [4,7]. Its synthesis is regulated by a complex sensing mechanism involving the HFE gene, Transferrin Receptor 2 (TfR2), and the BMP/SMAD signalling pathway, which monitors transferrin saturation and hepatic iron stores [4]. In states of iron sufficiency or systemic inflammation—driven largely by the IL-6/STAT3 signalling pathway—hepcidin levels rise significantly [4,5,30]. Hepcidin then binds to FPN on the surface of enterocytes and splenic macrophages, triggering the transporter’s internalisation and lysosomal degradation. This effectively halts iron entry into the plasma, leading to its sequestration within the reticuloendothelial system [7,31,32]. In chronic IMIDs, this mechanism often results in iron being present in body stores but unavailable for metabolic processes, contributing to the systemic burden of the disease.

### 2.2. Cellular Iron and Immune Function

The availability of intracellular iron is a fundamental determinant of immune cell fate, governing the transition from a quiescent state to an active, effector phenotype. In IMIDs, the local and systemic cytokine milieu significantly reconfigures cellular iron handling, which in turn modulates the intensity and duration of the inflammatory response.

#### 2.2.1. Cytokine-Mediated Regulation (IL-6, IL-1β, TNF-α)

In chronic inflammatory skin conditions, pro-inflammatory cytokines trigger nutritional immunity, which restricts iron to block pathogens but also reduces iron for the body’s immune response [12,14,16,33]. Interleukin-6 (IL-6) is the most potent inducer of hepatic hepcidin via the STAT3 signalling pathway; however, it also stimulates local hepcidin production within the skin and by infiltrating macrophages [7]. Tumor Necrosis Factor-alpha (TNF-α) and Interleukin-1beta (IL-1β) work synergistically in increasing iron sequestration through augmentation of ferritin (the iron-storing protein) and reduction in FPN expression on the cell surface [4,34,35]. This cytokine-induced “iron block” leads to an accumulation of intracellular iron within the reticuloendothelial system, a hallmark of the functional iron deficiency (FID) observed in patients with psoriasis and HS [4,6,36].

#### 2.2.2. Iron Dynamics in Macrophage Polarity and Lymphocyte Function

Iron serves as a metabolic switch for the polarisation of macrophages. M1 (pro-inflammatory) macrophages typically exhibit an “iron-retentive” phenotype, characterised by high levels of ferritin and low FPN, which supports the generation of antimicrobial reactive oxygen species (ROS) [12,14,23]. In contrast, M2 (anti-inflammatory/regulatory) macrophages favour an “iron-export” phenotype (high FPN, low ferritin), facilitating iron release into the microenvironment to support tissue repair and cellular proliferation [7,12].

T-cell activation is exceptionally sensitive to iron availability [13,14]. Upon antigen recognition, T-cells undergo rapid metabolic reprogramming, characterised by a dramatic upregulation of Transferrin Receptor 1 (TfR1/CD71) to internalise the iron required for mitochondrial biogenesis and DNA synthesis [13,15]. Recent studies have demonstrated that iron is essential for the stabilisation of Treg (regulatory T-cell) function; ID can impair Treg-mediated suppression by disrupting mitochondrial oxidative phosphorylation (OXPHOS), potentially exacerbating autoimmunity [12,16]. Furthermore, iron acts as an epigenetic modulator in T-cell differentiation [16]. Consequently, iron ID has been established as a critical factor in the impairment of Th17 and Th1 immune responses, leading to a marked reduction in the secretion of IL-17 and IFN-γ. In vitro evidence indicates that iron-deprived Th17 cells exhibit a significant decline in the expression of retinoic acid-related orphan receptor gamma t (RORγt)—the master transcription factor for this lineage—and a subsequent drop in IL-17a levels [12,33,36]. Similarly, experiments utilising iron chelators or iron-depleted media confirm that iron is indispensable to produce effector molecules in Th1 cells, most notably IFN-γ [12,14,36].

#### 2.2.3. Iron-Dependent Functions and Their Relevance to Chronic Skin Disease Mechanisms

**Figure 2:**
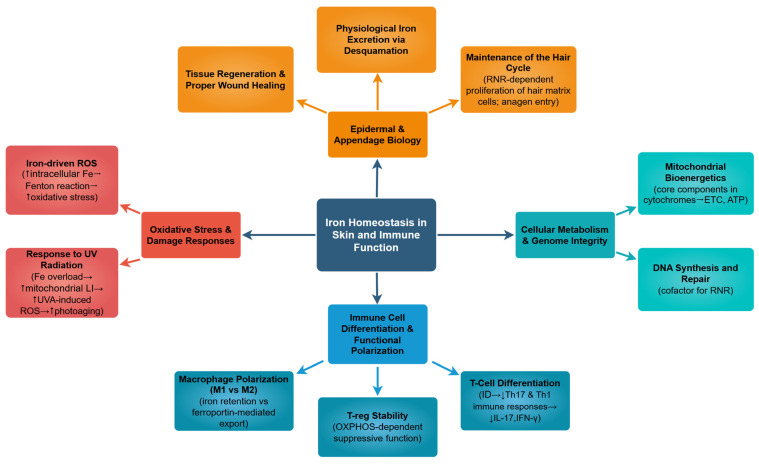
The Multifaceted Role of Iron in Cutaneous Homeostasis and Immunometabolism. Abbreviations: RNR—ribonucleotide reductase; ETC—electron transport chain; ROS—reactive oxygen species; LI—labile iron; OXPHOS—oxidative phosphorylation; ID—Iron deficiency; IL-17—Interleukin-17; IFN-γ—Interferon-gamma.

Iron is integral to a broad spectrum of cutaneous biological functions, linking mitochondrial metabolism, oxidative balance, and immune regulation with the maintenance of epidermal integrity, as schematically summarised in Figure 2. The total iron content in normal skin is estimated at 0.15–0.275 mg per gram of skin, with the highest levels present in the basal layer of the epidermis and progressively decreasing toward the stratum corneum [37].

Epidermal exfoliation constitutes a physiological pathway for cutaneous iron excretion, as iron incorporated into differentiating keratinocytes is ultimately lost with corneocytes during desquamation. In the basal layer of the epidermis, keratinocytes express TFR1, which mediates the uptake of circulating transferrin-bound ferric iron (Fe^3+^) [15]. Experimental studies have shown that overexpression of TFR1 enhances intracellular iron accumulation in both proliferating and differentiated keratinocytes, reflecting its key role in sustaining epidermal metabolism and regeneration [7,38]. Recent studies on senile lentigo (SL) further illustrate this dynamic; in SL lesions, the typical compartmentalization of iron proteins is lost. Molecules normally restricted to the lower epidermis—such as TFR1, Iron Regulatory Protein 1 (IRP1), and DMT1—show aberrant expression in the upper layers, while the iron exporter FPN is markedly diminished [28]. This process is accompanied by upregulated epidermal hepcidin expression, leading to an imbalance in iron metabolism that disrupts keratinocyte differentiation and melanin secretion [39]. More importantly, epidermal TFR1 dysregulation is implicated in cutaneous oncogenesis, showing profoundly upregulated TFR1 expression in malignant transforms of epidermal cells compared with benign epidermal melanocytic nevi [21]. TFR1 is thus clearly recognised as an important molecular factor in epidermal iron metabolism, finely balancing iron uptake, storage, and loss.

Recent findings revealed that the epidermis sustains an intricate iron recycling system mediated by HO-1, FPN, and hephaestin-like 1 (HEPHL1), ensuring the conservation of iron during keratinocyte differentiation and exfoliation, as well as minimising elemental loss from the stratum corneum [13,17,40]. HO-1, regulated by Nrf2, catalyses heme degradation to generate Fe^2+^, biliverdin, and carbon monoxide (CO), thereby supporting antioxidant defence in differentiated keratinocytes [40]. Nrf2-dependent upregulation of HO-1 has been demonstrated in inflammatory skin diseases such as psoriasis [17]. Moreover, exposure to long-wave UVA1 radiation induces HO-1 expression, indicating that this pathway contributes to photoprotective, antioxidant, and anti-inflammatory responses in the skin [18]. FPN is abundantly expressed in the granular layer of the epidermis, where it transports Fe^2+^ ions from keratinocytes to the extracellular space [19]. Abrogation of SLC40A1 gene expression (encoding FPN) in human keratinocytes enhances cellular iron content, which further verifies its involvement in iron export from the epidermis [7,32,34]. The iron content is increased twofold within the stratum corneum in keratinocyte-selective FPN knockout mice. Notably, when fed low iron diet, these mice also develop anaemia and reduced body weight, indicating that skin FPN is involved in iron homeostasis, both locally and systematically [18,19]. HEPHL1 is a transmembrane ferroxidase homologue that is preferentially expressed within the suprabasal epidermis, oxidising iron from the reduced, ferrous state (Fe^2+^) to the oxidised, ferric state (Fe^3+^), which is then suitable for its integration within transferrin [41,42]. Although human HEPHL1 is expressed moderately within the skin, it is abundantly expressed within pilosebaceous units, which promote epithelial integrity and hair development, respectively [42,43,44].

**Figure 3:**
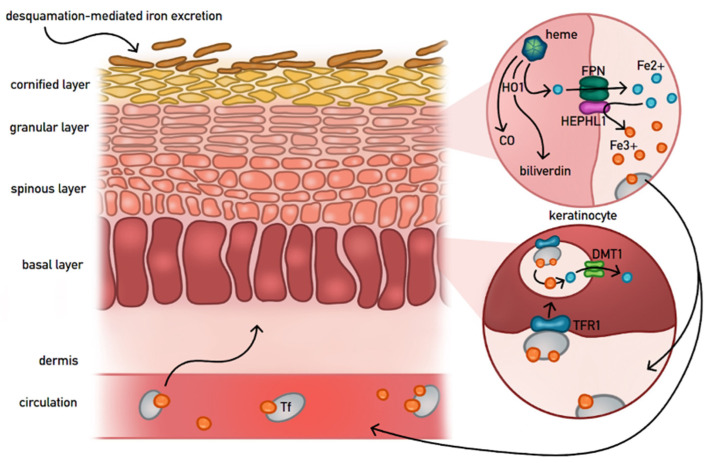
Epidermal iron homeostasis. Schematic representation of iron uptake, recycling, export, and physiological loss during keratinocyte differentiation. Basal keratinocytes acquire transferrin-bound iron via TFR1, while suprabasal and granular layers coordinate iron recycling through HO-1, FPN, and HEPHL1, limiting elemental loss during epidermal turnover [7,13,15,19,38].

Figure 3 presents a schematic overview of epidermal iron homeostasis, integrating the mechanisms described above.

Iron acts as a fundamental bioenergetic catalyst in the skin, where it is indispensable for mitochondrial respiration and DNA synthesis in both keratinocytes and fibroblasts. By serving as a critical cofactor for ribonucleotide reductase, iron facilitates the production of deoxyribonucleotides required for genomic replication, thereby supporting robust cellular proliferation and tissue regeneration [23,25,45]. Furthermore, within the mitochondria, iron is incorporated into iron-sulfur (Fe-S) clusters and heme groups of the cytochromes [23]. These components are essential for the electron transport chain, maintaining the high bioenergetic demands required for the activation and effector functions of skin-resident immune cells [23,45,46]. Recent studies confirmed that UVA-induced oxidative stress is amplified under iron overload, accelerating photoaging and keratinocyte apoptosis through mitochondrial dysfunction [25,47,48]. This is particularly evident in skin fibroblasts from Friedreich’s ataxia (FRDA) patients, which exhibit a 4- to 10-fold higher sensitivity to UVA-induced death than healthy cells. Research shows that elevated mitochondrial labile iron (LI) catalyses a surge in ROS upon UVA exposure, leading to mitochondrial membrane disruption and necrotic cell death through ATP depletion. Crucially, pre-treatment with mitochondrial-targeted iron chelators can fully abrogate this damage, identifying mitochondrial LI as a key therapeutic target for photoprotection [25].

Beyond its role in energy metabolism, iron is a key modulator of melanogenesis and skin pigmentation, primarily through its regulation of tyrosinase, the rate-limiting enzyme in melanin biosynthesis [49,50]. Tyrosinase requires copper as a cofactor, but iron indirectly influences its activity by regulating redox balance, melanosomal maturation, and melanocyte viability [50]. Optimal intracellular iron homeostasis is therefore essential for normal melanin synthesis, while deviations in iron levels—either deficiency or overload—can disrupt pigmentation patterns. Conversely, iron overload promotes hyperpigmentation and oxidative damage. In hereditary and secondary hemochromatosis, excessive iron accumulation in the skin results in melanoderma—a greyish or bronze discoloration caused by both hemosiderin deposition and increased epidermal melanin [51,52]. Histopathological analyses show that hemosiderin, an iron-storage complex derived from partially degraded ferritin and lysosomes, accumulates in dermal macrophages and perivascular regions, contributing to diffuse hyperpigmentation [29,53]. Early experimental studies confirmed a causal link between cutaneous iron deposition and pigmentation, where parenteral iron administration in mice induced pronounced dermal iron accumulation, enhanced hemosiderin formation, and darker skin tones [54]. Similarly, iron loading in cultured retinal pigment epithelial cells increased melanin synthesis, demonstrating that iron directly upregulates melanogenic pathways [55]. Interestingly, not all pigmentary disorders associated with iron involve systemic iron excess. For instance, individuals with melasma—a common acquired hypermelanosis—exhibited lower serum iron levels compared to controls [56]. This suggests that local iron metabolism in the skin may differ from systemic iron homeostasis, with tissue-specific iron sequestration or redistribution potentially influencing melanocyte function and local oxidative signalling.

### 2.3. The Iron–Skin Axis

The skin is increasingly recognised as a dynamic regulator of iron metabolism rather than a passive site of deposition or loss [13]. A recent study demonstrated that keratinocytes directly sense and respond to systemic iron levels via ferritin-mediated sequestration and transferrin receptor regulation, linking epidermal iron storage to systemic hepcidin feedback. Upon iron overload, ferritin heavy chain (FTH1) expression increases, while iron chelation by deferoxamine reduces both ferritin and cuproenzyme ceruloplasmin, underscoring a coordinated response to maintain redox equilibrium [22]. Epidermal desquamation thus serves as a unique route for iron excretion, and excessive ferritin loss during chronic inflammation may underlie iron-deficiency anaemia observed in exfoliative dermatoses and psoriasis [6,17,20,57].

Altogether, the iron–skin axis operates as an integrated network balancing cellular iron flux, redox homeostasis, and immune signalling. Its dysregulation links molecular iron handling with dermatologic pathophysiology—from barrier dysfunction and pigmentation anomalies to photoaging and immune-mediated inflammation—positioning iron metabolism as a central therapeutic target in cutaneous medicine [35,40,42,50].

## 3. Iron Deficiency and Systemic Inflammation: The Dermatological Perspective

### 3.1. Anaemia of Inflammation as a Systemic Consequence of Chronic Immune Activation

Patients with chronic inflammation may develop disturbances of iron homeostasis, leading to hypoferremia and iron-restricted erythropoiesis. This process may result in the development of a condition known as anaemia of inflammation (AI). It affects key cellular functions, leading to reduced oxygen transport, impaired energy production, and diminished cell proliferation, which ultimately impacts tissue function and overall health [58,59,60,61,62].

The spectrum of conditions in which chronic inflammation disrupts iron homeostasis is broad. These phenomena are particularly relevant in IMIDs affecting the skin, such as psoriasis and atopic dermatitis [6,11,58].

Patients with anaemia associated with chronic inflammatory diseases typically present with normocytic, normochromic, hypoproliferative anaemia of mild to moderate severity (haemoglobin 8–10 g/dL). It is characterised by elevated inflammatory markers (CRP, ESR, IL-6), low serum iron and transferrin saturation, and iron sequestration within macrophages, resulting in functionally unavailable iron and often normal or elevated serum ferritin [32,57,63]. However, ferritin concentrations in inflammatory states reflect both increased ferritin production by iron-retaining macrophages and its role as an acute-phase reactant induced by inflammatory mediators. Consequently, its levels may not accurately reflect body iron stores in inflammatory settings and cannot exclude coexisting absolute iron deficiency [58]. AI may be resistant to iron therapy, and treatment focuses on the underlying disease, though it is not always effective, and these disturbances can also impact tissues with high proliferative and metabolic demands, including the skin [64].

### 3.2. Molecular Mechanisms of Iron Sequestration: The IL-6-Hepcidin-Ferroportin Axis

#### 3.2.1. Immunopathogenesis of Anaemia of Inflammation and Functional Iron Deficiency

The pathogenesis of iron dysregulation in IMIDs is complex and arises from immune modulation associated with the underlying condition. Chronic immune activation and elevated levels of proinflammatory cytokines, primarily IL-6, lead to FID, in which, despite normal or increased body iron stores, the amount available for erythropoiesis is limited, resulting in the development of AI.

This process involves three primary pathophysiological pathways: iron sequestration in macrophages, inflammatory suppression of erythropoiesis, and shortened erythrocyte survival. The IL-6–hepcidin–ferroportin axis plays a central role in coordinating the transition from systemic inflammation to FID. Other factors contributing to systemic anaemia—including impaired erythropoietic response, altered erythrocyte dynamics, and compensatory hepcidin regulation via the EPO–erythroferrone axis—also play important roles; however, a detailed discussion of these iron-independent pathways falls beyond the scope of this skin-focused review [32,58,61,63,65,66].

These changes extend beyond the hematopoietic system, causing systemic metabolic disturbances relevant for other organs, including the skin [67].

#### 3.2.2. Hepcidin-Mediated Iron Sequestration

Systemic inflammation triggers immune activation and induces a signalling cascade in which proinflammatory cytokines, such as IL-6 and IL-1β, as well as lipopolysaccharide (LPS), stimulate hepatocytes to synthesise hepcidin. At the same time, the expression of transferrin, the primary iron transport protein, is reduced [58,68,69].

IL-6 enhances JAK/STAT signalling by promoting STAT3 phosphorylation and its binding to the hepcidin gene promoter, thereby increasing hepcidin transcription. IL-1β and IL-22 can also stimulate hepcidin synthesis. The elevated hepcidin production observed in systemic inflammatory diseases reflects its role as a key mediator of innate immunity [30,32,58,64,68,70].

Increased circulating hepcidin levels lead to its binding to the only known transmembrane exporter, FPN, causing its internalisation and degradation. This decreases FPN expression in macrophages of the mononuclear phagocyte system (MPS) and duodenal enterocytes, leading to iron sequestration in macrophages and reduced dietary iron absorption, thereby limiting iron availability for erythropoiesis. Iron retention in macrophages is particularly important because iron recycled from senescent erythrocytes by the MPS provides over 90% of the daily iron required for haemoglobin synthesis and erythropoiesis [32,58,63,68].

Proinflammatory cytokines, including IL-1β, IL-6, IL-10, and IFN-γ, increase iron uptake by macrophages and promote its efficient storage by inducing ferritin, the primary iron storage protein characteristic of inflammation. At the same time, they restrict iron export by suppressing FPN expression and enhancing free radical-mediated damage to erythrocytes and their phagocytosis. The synergistic action of these mechanisms results in limited iron availability for erythropoiesis, leading to the hypoferremia and hyperferritinemia typical for inflammatory anaemia [58,66,71].

### 3.3. Cutaneous Consequences of Functional Iron Deficiency

ID is a condition in which the availability of iron is insufficient to meet the body’s metabolic needs and can occur with or without anaemia. This deficiency causes a range of nonspecific symptoms, which often appear before the development of anaemia [72].

Iron homeostasis in the skin is regulated by complex mechanisms, including the hepcidin-ferroportin pathway, which controls iron import, storage, recycling, and release, thereby maintaining its balance in skin tissue [22]. Its balance is essential for proper skin function, and dysregulation can exacerbate pathologies observed in chronic inflammatory skin diseases. Disturbances in iron availability, such as FID occurring during chronic inflammation, may adversely affect cells with high proliferative capacity and metabolic demand, including keratinocytes. Iron is required for numerous key cellular processes, including mitochondrial respiration, DNA synthesis, and detoxification reactions, as well as for the proper proliferation and differentiation of epithelial cells, which is crucial for skin function and regeneration [11,13,22,57,67,73].

#### 3.3.1. Mitochondrial Dysfunction as a Mechanistic Consequence of Iron Deficiency

In the skin, mitochondria play a central role in energy production, regulation of oxidative stress, and the biosynthesis and protection of tissue. They support crucial skin functions by promoting keratinocyte differentiation, fibroblast collagen synthesis, and melanocyte pigment production, while protecting against UV-induced oxidative damage and maintaining oxidative balance. Adequate ATP production drives cell division, repair, and regeneration, all of which are essential for proper cellular function and the maintenance of skin integrity [24,45,46,74,75].

Mitochondria are also central hubs of iron metabolism, requiring adequate iron availability for proper function. Iron is incorporated into heme- and iron–sulfur-containing proteins of the respiratory chain, and its deficiency impairs mitochondrial activity, leading to reduced ATP production and altered redox balance. Cells with high proliferative and metabolic demands, including keratinocytes, fibroblasts, and melanocytes, are particularly sensitive to these disturbances [23,24,46,75].

**Figure 4:**
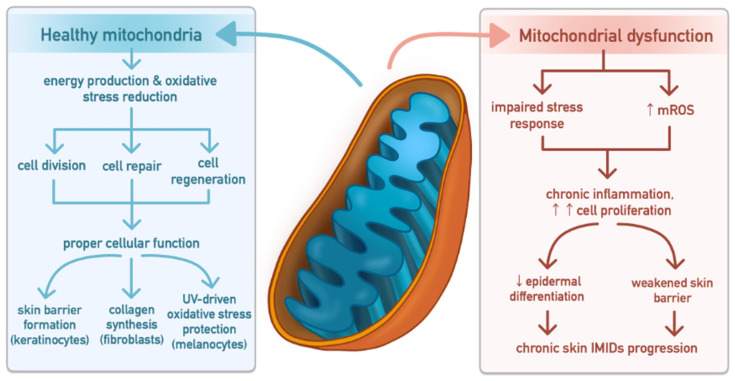
Mitochondrial integrity and dysfunction in skin homeostasis and disease [24,45,46,74,75]. Mitochondria regulate ATP production, redox balance, and biosynthetic pathways essential for skin function. In keratinocytes, they support differentiation and barrier formation; in fibroblasts, collagen synthesis and oxidative balance; and in melanocytes, melanin production and protection against UV-induced oxidative stress. Dysregulation of mitochondrial biogenesis and dynamics leads to mitochondrial oxidative stress, impaired stress responses, and disrupted cellular homeostasis. Persistent mitochondrial dysfunction promotes chronic inflammatory signalling, aberrant proliferation, defective epidermal differentiation, and barrier disruption, contributing to progression of chronic immune-mediated inflammatory skin diseases (IMISDs). Abbreviations: mROS—mitochondrial reactive oxygen species; IMIDs—immune-mediated inflammatory diseases.

Dysregulation of mitochondrial biogenesis and dynamics culminates in skin cell mitochondrial dysfunction, impairing stress responses and disrupting cellular homeostasis. As illustrated in Figure 4, the transition from healthy mitochondrial networks to dysfunctional mitochondria is accompanied by excessive mitochondrial oxidative stress, which drives cutaneous inflammation, tissue damage, and aberrant cell proliferation. Persistent mitochondrial dysfunction further compromises epidermal differentiation and skin barrier integrity, sustaining chronic inflammatory signalling and contributing to the initiation and progression of chronic immunologically mediated inflammatory skin diseases, including psoriasis and AD [24,45,46,74,75].

#### 3.3.2. Iron and Skin Regeneration: Impaired Wound Healing

Cutaneous wound healing is a dynamic and complex process involving cellular, humoral, and molecular mechanisms. Various cell types participate in the repair of damaged skin, including keratinocytes, fibroblasts, and innate immune cells, particularly macrophages [22,67].

Iron is considered a crucial element of this process, and studies have shown that chronic ID can compromise repair through various mechanisms, leading to reduced wound strength, impaired regeneration, and delayed skin healing. An important modulator of healing is lactoferrin, an iron-binding glycoprotein that supports the initial inflammatory phase, stimulates cell proliferation and migration, and prevents wounds from becoming chronic [22,76].

During the wound healing process, iron is essential for collagen synthesis, the main component of the extracellular matrix, which is produced primarily by fibroblasts. Collagen provides structural integrity and mechanical strength to tissues and serves as a substrate for cell adhesion, proliferation, and differentiation, thereby supporting the repair and regeneration of damaged tissues, particularly during the wound remodelling phase [76,77]. Adequate iron levels are also required for efficient oxygen utilisation in proliferation, bacterial defence, and angiogenesis [76].

Impaired wound healing in inflammatory diseases may result from both systemic anaemia and disturbances in local iron homeostasis in the skin [22,67]. Under inflammatory conditions, elevated hepcidin levels cause pathological iron retention in macrophages by inactivating FPN [22]. This results in delayed healing, impaired granulation tissue formation, and impaired fibrogenesis and angiogenesis [76].

Iron-overloaded macrophages exhibit enhanced release of TNF-α and ROS, including hydroxyl radicals, which contribute to the persistence of chronic inflammation. The sustained proinflammatory phenotype of these cells induces a p16INK4a-dependent cellular senescence program in resident skin fibroblasts. This leads to G1 phase cell cycle arrest, consequently impairing wound regeneration and hindering proper healing [22,78].

All the processes described, from disturbances in iron homeostasis and lactoferrin function, to reduced collagen synthesis, disrupted cell proliferation and angiogenesis, and chronic inflammation induced by iron retention in macrophages, may result from dysregulated iron levels and contribute to compromised skin wound healing in chronic IMIDs [22,37,67].

## 4. Iron Deficiency in Immune-Mediated Inflammatory Skin Diseases (IMISDs)

### 4.1. Psoriasis

A recent retrospective cohort study involving 156 million patients investigating vitamin deficiencies and laboratory abnormalities in psoriasis demonstrated a significantly higher prevalence of ID among patients with psoriasis compared to the control group [79]. Although chronic inflammation in psoriatic patients may cause ID, low hepcidin levels suggest it is due to depleted iron stores rather than inflammation-driven deficiency [6]. Recent research supports this hypothesis, suggesting that patients with exfoliative psoriasis may develop ID due to high skin iron levels and daily losses of up to 8 mg, indicating cutaneous sequestration as a cause [38]. This paradigm differs from traditional models of anaemia in chronic disease. In particular, hepcidin, an iron-regulatory hormone, is overexpressed in patients with psoriasis and causes iron accumulation in keratinocytes. This process initiates an inflammatory cascade that promotes epidermal ferritin accumulation, hyperproliferation and elevates CXCL1 production, ultimately facilitating neutrophil recruitment [22,38]. Clinical data indicate that iron dysregulation in psoriasis is affected by metabolic status. Ponikowska et al. found no association between psoriasis severity and iron biomarkers, but patients with a BMI under 24 kg/m^2^ had lower ferritin, lower hepcidin, and a higher incidence of ID (82% vs. 39%), suggesting a cachexia-like mechanism in these cases [6].

El-Rifaie et al. in their study, measured HO-1 expression alongside iron biomarkers. Their findings were consistent with other studies, demonstrating reduced serum iron, hepcidin and TIBC, accompanied by elevated soluble transferrin receptor levels [80]. In addition, HO-1 gene expression was significantly upregulated. Heme oxygenase is integral to iron metabolism, catalysing the breakdown of heme to liberate iron. The predominant source of iron for heme synthesis is obtained via the recycling of heme from aged erythrocytes through this enzymatic pathway. As HO-1 is induced under conditions of ID, its overexpression may represent a compensatory response aimed at mobilising iron to meet the demands of heme synthesis [80].

### 4.2. Atopic Dermatitis

AD dis a complex and multifactorial condition that involves changes in both humoral and cell-mediated immune responses, as well as disturbances in the skin’s barrier and inflammatory processes [81]. Recent studies have demonstrated that patients with AD show significant reduction in iron levels in peripheral blood and serum as well as decreased transferrin saturation and lower ferritin levels [11,82]. In a study by Ponikowska et al., systemic ID was significantly associated with greater impairment in Dermatology Life Quality Index (DLQI) scores, while elevated transferrin levels correlated positively with increased disease severity as measured by Eczema Area and Severity Index (EASI) and SCORing Atopic Dermatitis (SCORAD) indices [11]. In the study, 45% of individuals with AD had low transferrin saturation (Tsat), 37% had low ferritin, and 26% showed reduced serum iron. Notably, patients with evidence of pro-inflammatory activation (measured by CRP levels) exhibited a pattern of lower iron and Tsat accompanied by elevated sTfR levels [11].

These clinical associations are supported by immunological evidence demonstrating the impact of ID on regulatory immune pathways in AD. ID was shown to affect CD24^+^CD38^+^CD19^+^ regulatory B cell (Breg) function and reduce IL-10 production. These mechanisms constitute essential elements of humoral immune regulation in AD [82]. A positive correlation was identified between serum iron concentrations and IL-10 levels, suggesting that iron status may influence the functional activity of Breg cells. When iron levels were reduced using Ciclopiroxolamine, IL-10 expression also decreased. These findings suggested that ID may play a role in disrupting immune balance and worsening inflammation in AD [82]. Although mechanistic studies are limited, cutaneous iron buildup and IL-36 involvement in AD suggest that hepcidin-driven iron retention may promote skin inflammation, as seen in psoriasis [22,81].

Impaired immune regulation, particularly reduced IL-10 levels and weakened skin defences, may foster an environment conducive to microbial imbalance, promoting the overgrowth of *Staphylococcus aureus* and other microorganisms commonly associated with AD [83]. In response to microbial invasion, host cells release iron-binding proteins such as lactoferrin and calprotectin to restrict microbial access to essential nutrients—a defence mechanism known as nutritional immunity [84]. However, *Staphylococcus aureus* utilises siderophores (staphyloferrins), high affinity iron chelators along with the Isd heme uptake pathway to extract heme from inflamed skin tissue, making these defence mechanisms less effective [85,86]. This ability to survive in environments that are both low in iron and inflamed not only helps bacteria persist, but may also further disturb the host’s iron balance, leading to increased oxidative stress and heightened inflammatory responses [11,85].

### 4.3. Hidradenitis Suppurativa

HS is a chronic inflammatory disease characterised by recurrent, deep-seated nodules, abscesses, and fistulae mostly affecting the axillary, inguinal, and submammary regions [87]. Although the pathogenesis of HS is not yet fully understood, immune dysregulation mechanisms play an important role. Dysregulation involving the Th1/Th17 axis and increased cytokine release, particularly TNF-α and IL-1β, leads to systemic inflammation and may contribute to the development of ID and anaemia of chronic disease [88,89]. In a comparative study involving 74 patients with HS and 44 healthy controls, several iron metabolism parameters were evaluated, such as ferritin, Tsat, soluble transferrin receptor, and hepcidin. Patients with HS showed significantly lower levels of ferritin, Tsat, and hepcidin, with ID present in 75% of the HS group. Notably, iron abnormalities were not associated with disease severity and did not correlate with IL-6 levels [36]. Researchers speculate that the mechanisms in HS differ from other chronic inflammatory conditions because patients exhibit low hepcidin levels, which may also point to an impairment in the body’s innate antimicrobial defence systems. Furthermore, the lack of correlation between ID biomarkers and IL-6 or obesity suggests that this deficiency might be driven by unique, non-inflammation-driven pathways that require further investigation to determine whether they offer a potential therapeutic target [36].

In another retrospective cohort study including 2364 patients with HS, it was confirmed that HS is strongly associated with several types of anaemia. Anaemia was present in 18.4% of patients with HS compared with 3.4% in the general hospital population. Patients with HS had significantly higher odds of IDA, anaemia of chronic disease, and sickle cell anaemia [90].

A retrospective study conducted by Resnik et al., encompassing 92 patients with HS, found an even higher prevalence of anaemia of 41.3%, exceeding rates reported in prior studies. The study identified male sex and African American race as independent risk factors. Results showed that men are four times more likely, and African American patients are over three times more likely to be affected [91]. These findings underscore the need for further research to fully define independent risk factors and to develop targeted screening strategies.

### 4.4. Lupus Erythematosus

The pathophysiology of lupus erythematosus (LE) is based on a persistent proinflammatory state and ongoing immune activation. Emerging evidence suggests that the pathological redistribution of iron is triggered by chronic immune dysregulation, involving the overproduction of TNF-α and IL-6 [92]. To explore how iron-related biomarkers reflect disease status, a study of 28 individuals with SLE measured transferrin and ferritin levels in both serum and urine [93]. Ferritin concentrations in both urine and serum were elevated in individuals with active SLE and showed a positive association with IL-6 and TNF-α levels; however, these correlations did not reach statistical significance. Notably, ferritin levels in both compartments also correlated with anaemia in SLE patients. A similar trend was observed for urinary transferrin. However, serum transferrin levels were reduced in patients with active SLE. Together, these findings reflect what researchers describe as an “ominous triad” of systemic inflammation, elevated ferritin, and anaemia of chronic disease [93].

At the molecular level, recent mechanistic studies have uncovered a previously unrecognised role for the RNA-binding protein Pumilio2 (Pum2) in LE pathogenesis [94]. Dysregulated Pum2 activity leads to intracellular iron accumulation. This excess iron amplifies macrophage-mediated inflammatory responses. Through the Pum2-dependent pathway, RNA regulation becomes directly tied to iron balance and innate immune signalling. This connection clarifies why persistent IgG immune complexes can escalate inflammation in LE [94]. In addition to systemic immune dysregulation, environmental factors such as ultraviolet (UV) radiation can further disturb iron homeostasis at local tissue sites. In cutaneous manifestations of LE, UV exposure may trigger localised iron release within the skin, which in turn impairs the anti-inflammatory function of the Ro52 antigen—an antigen that is notably upregulated in the keratinocytes of photosensitive patients [67].

While ID is well-documented in systemic lupus erythematosus (SLE), affecting approximately 36% of anaemic SLE patients [95,96], there is no direct evidence examining ID in cutaneous lupus erythematosus (CLE) populations. This gap is particularly problematic given that it may have different risk profiles for ID. The established impact of UV radiation suggests that iron dysregulation may also contribute to the pathogenesis of CLE. However, the absence of direct clinical and mechanistic studies in CLE limits the ability to determine how iron status influences the course of the disease.

### 4.5. Other Immune-Mediated and Barrier-Disruptive Skin Disorders

While iron dysregulation is well documented in LE, HS, AD, and psoriasis, new findings suggest it may also influence other immune-mediated skin diseases. In a large case–control study, 588 patients with oral lichen planus (OLP) had significantly higher rates of haematological abnormalities. Deficiencies in haemoglobin, serum iron, vitamin B12, and folic acid were significantly more common in OLP individuals compared with matched healthy controls. Notably, anaemia was present in 25.2% of individuals with OLP, with FID accounting for 21.6% of cases. These findings were accompanied by elevated rates of serum gastric parietal cell antibody (GPCA), indicating possible autoimmune gastritis and malabsorption contributing to micronutrient deficiencies [97]. In another study involving 352 patients with OLP, ID was identified in 13.6% of individuals, and anaemia was present in 21.9%—both significantly higher than in healthy controls [98]. These findings further support an association between OLP and iron dysregulation across multiple cohort studies.

Epidermolysis bullosa (EB) represents another condition in which ID and anaemia are highly prevalent. In a cohort of 169 patients with EB, anaemia was identified in 27.8% of individuals. The most severe subtypes—recessive dystrophic EB and generalised severe junctional EB—showed anaemia rates of 100% and 68%, respectively [99]. An overview of IMISDs, including disease prevalence, alterations in iron biomarkers, proposed underlying mechanisms, and clinical implications, is provided in Table 1.

## 5. Therapeutic Considerations and Future Directions

A growing body of evidence suggests that disturbances in iron metabolism and homeostasis, including AI, represent a significant, yet underexplored component of chronic IMISD progression and development. Disruption of systemic and local iron homeostasis in chronic inflammatory skin diseases impairs epidermal function and promotes inflammation, highlighting the iron–skin axis as a promising therapeutic target [22,38,100]. Future research efforts should move beyond associative observations and focus on bridging the underlying molecular mechanisms with possible clinical interventions in dermatology.

Relevance and impact assessment of iron supplementation in skin IMIDs, including comparison between oral and intravenous administration, remains one of the key directions of investigation. In conditions of chronic inflammation, correlated with elevated hepcidin levels and FID, the efficacy of both types of iron therapy can be limited. Available data, stemming primarily from studies of various IMIDs, indicate that neither oral nor intravenous iron treatment provides significant improvements in iron status markers or clinical outcomes [101]. Experimental evidence from rat models further suggests that in AI, increased inflammatory activity limits the efficacy of both oral and intravenous iron, with intravenously administered iron becoming trapped in macrophages and potentially aggravating inflammation [102]. Thus, clinical trials designed to identify dermatological patient subgroups likely to benefit from therapy, together with therapeutic strategies that bypass the hepcidin-dependent blockage of iron availability, remain essential.

In parallel, a promising area of research involves interventions aimed at modulating the hepcidin-ferroportin axis. Pharmacological advances enable the development of new therapeutic strategies, encompassing hepcidin modulators and FPN agonists. These approaches either directly target these molecules or act on upstream regulatory pathways, including inflammatory signalling cascades that control their expression.

Hepcidin antagonism has been proposed as a potential strategy to promote iron mobilisation from stores and enhance its availability for erythropoiesis in iron-restrictive anaemias. Beyond the systemic effects, emerging evidence suggests that in inflammatory skin diseases such as psoriasis, keratinocyte-derived hepcidin plays a role in disease pathogenesis through promoting epidermal hyperproliferation and neutrophil recruitment. This supports the rationale for consideration of hepcidin-directed therapeutic approaches in dermatology. While multiple classes of hepcidin-targeted agents are under development, their clinical applicability and safety remain to be established [22,31,64].

FPN agonists represent an alternative strategy to mitigate hepcidin-driven iron restriction by stabilising the transporter and maintaining iron export. Although several FPN agonists or stabilising agents have been identified in preclinical studies, their therapeutic potential in inflammatory disease settings is yet to be defined [31].

Collectively, current evidence indicates that conventional iron supplementation has limited efficacy in inflammatory settings, while therapeutic modulation of the hepcidin–ferroportin axis represents a promising, yet still mostly unvalidated strategy. These observations underscore the need for further research to clarify how systemic and local iron regulation influences inflammatory activity and, therefore, disease course in chronic IMISDs. Well-designed translational and clinical studies remain essential to determine whether targeting the iron-skin axis can improve patient outcomes.

## 6. Materials and Methods

A comprehensive literature search was conducted using databases such as PubMed, Google Scholar, and Scopus, focusing primarily on studies published between 2015 and 2025; however, earlier pivotal publications were also included when deemed relevant. Additional sources such as Cochrane Library and ClinicalTrials.gov were used. “iron deficiency”, “iron metabolism”, “functional iron deficiency”, “immune-mediated inflammatory skin diseases”, “psoriasis”, “atopic dermatitis”, “hidradenitis suppurativa”, “systemic inflammation”, “immunometabolism”, “hepcidin”, “ferroportin”, “keratinocytes”, and “iron–skin axis”. While advanced search methods prioritised literature from the past five years, this review analyses research spanning from 1999 to 2025. Figure 1, Figure 2, Figure 3 and Figure 4 were created using diagrams.net (formerly draw.io), web app version 14.6.13 (accessed on 20 December 2025), and Autodesk Sketchbook for iPad, version 6.2 (released 23 May 2025)—all based on the authors’ interpretation of synthesised data from multiple studies.

## 7. Conclusions

ID and disrupted iron homeostasis represent a crucial yet still underrecognized factor in the pathogenesis of IMISDs associated with chronic systemic inflammation. Persistent inflammatory activation, primarily driven by the IL-6-hepcidin-ferroportin axis, reduces iron bioavailability, induces FID, and impairs mitochondrial function, immune regulation, epidermal differentiation, and tissue repair.

The skin actively participates in iron metabolism, with keratinocyte-mediated iron uptake and storage significantly shaping the local inflammatory environment. Disruption of these processes enhances oxidative stress, increases susceptibility to ferroptosis, impairs epidermal barrier function, and sustains immune activation, thereby contributing to disease chronicity and the emergence of systemic symptoms such as fatigue and reduced physical performance. Clinical data across multiple IMISDs consistently indicate a high prevalence of ID and anaemia, along with disease-specific patterns of regulatory dysfunction.

These findings position iron metabolism as a key pathophysiological factor that should be systematically considered in the assessment of patients with chronic inflammatory skin diseases. The limited effectiveness of conventional iron supplementation in inflammatory conditions highlights the need for biomarker-guided patient stratification and the development of therapeutic strategies targeting the hepcidin-ferroportin axis. Interventions aimed at the iron-skin axis may represent a promising approach to improving disease control and reducing systemic disease burden, but more translational and clinical studies are needed.

## Figures and Tables

**Table 1:** Overview of Iron Dysregulation Across Immune-Mediated Inflammatory Skin Diseases (IMISDs).

Disease	Reported Prevalence of ID/Anaemia	Iron Biomarker Profile	Proposed Mechanisms	Clinical Impact	Risk Modifiers	References
Psoriasis	Significantly higher than controls; 82% in patients with BMI < 24	Variable ferritin/hepcidin patterns; lower ferritin and lower hepcidin in cachexia phenotype; higher ferritin and higher hepcidin in inflammatory phenotype; higher HO-1; higher sTfR	Cutaneous iron loss, keratinocyte sequestration, hepcidin-driven inflammation	Limited direct data on patient-reported outcomes; ID reported in inflammatory and low-BMI phenotypes	Lower BMI (<24 kg/m^2^); inflammatory disease activity	[6,22,38,79,80]
Atopic Dermatitis (AD)	45% low Tsat; 37% low ferritin; 26% low serum iron	Lower serum iron, lower ferritin, lower Tsat; higher transferrin; higher sTfR; lower IL-10	Impaired Breg/IL-10 function, microbial dysbiosis, nutritional immunity failure	ID correlates with worse DLQI; high transferrin correlates with disease severity (EASI/SCORAD)	Higher disease severity	[11,22,86,87,88,89,90,91]
Hidradenitis Suppurativa (HS)	75% ID; 18.4–41.3% anaemia (higher than general population)	Lower ferritin, lower Tsat, lower hepcidin; normal IL-6	Non-IL-6 inflammation; impaired iron regulation, racial/sex disparities	Limited data on patient-reported outcomes directly attributable to ID	Male sex (~4× risk); African American race (~3× risk); severe disease	[36,93,94,95,96]
Lupus Erythematosus (LE)	ID in 36% of anaemic patients	Higher ferritin (serum & urine), lower serum transferrin, higher urinary transferrin	TNF-α/IL-6–associated iron sequestration; Pim2-related pathways; inflammation-associated redistribution	ID observed in active SLE; overlaps with anaemia of chronic inflammation	Active systemic disease	
Oral Lichen Planus (OLP)	13.6–25.2% anaemia; 21.6% of those cases are ID	Lower iron, lower B12, lower folate; higher GPCA	Autoimmune gastritis, malabsorption, micronutrient deficiency	—	Higher prevalence of haematologic abnormalities compared with controls	[97,98]
Epidermolysis Bullosa (EB)	27.8% overall; 100% in recessive dystrophic EB; 68% in generalised severe junctional EB	Lower Hb; ID frequently reported in severe subtypes	—	—	Higher prevalence in severe subtypes	[99]

Abbreviations: ID—iron deficiency, IMISDs—immune-mediated inflammatory skin diseases, BMI—body mass index, HO-1—heme oxygenase-1, sTfR—soluble transferrin receptor, AD—atopic dermatitis, Tsat—transferrin saturation, IL-10—interleukin-10, Breg—regulatory B cells, DLQI—Dermatology Life Quality Index, EASI—Eczema Area and Severity Index, SCORAD—Scoring Atopic Dermatitis, HS—hidradenitis suppurativa, IL-6—interleukin-6, LE—lupus erythematosus, SLE—systemic lupus erythematosus, TNF-α—tumour necrosis factor alpha, OLP—oral lichen planus, B12—vitamin B12, GPCA—gastric parietal cell antibodies, EB—epidermolysis bullosa, Hb—haemoglobin.

## Data Availability

No new data were created or analysed in this study. Data sharing is not applicable to this article.
